# Novel Biomarkers and the Future Potential of Biomarkers in Inflammatory Bowel Disease

**DOI:** 10.1155/2017/1936315

**Published:** 2017-04-16

**Authors:** Gilles Duvoisin, Robert N. Lopez, Andrew S. Day, Daniel A. Lemberg, Richard B. Gearry, Steven T. Leach

**Affiliations:** ^1^Department of Paediatrics, Lausanne University Hospital and University of Lausanne, Lausanne, Switzerland; ^2^Department of Gastroenterology, Sydney Children's Hospital Randwick, Sydney, Australia; ^3^Department of Medicine, University of Otago, Christchurch, Christchurch, New Zealand; ^4^Department of Paediatrics, University of Otago, Christchurch, Christchurch, New Zealand; ^5^School of Women's and Children's Health, University of New South Wales, Sydney, NSW, Australia

## Abstract

There is increasing importance placed upon noninvasive assessment of gut inflammation. These tools are likely to be the key in differentiating intestinal inflammatory disease from functional disorders and in monitoring the response to intervention in individuals with known inflammatory conditions. Although various noninvasive markers are currently available, they have limitations and do not provide ideal utility. This review focuses on emerging markers of gut inflammation, highlighting the potential of specific markers.

## 1. Introduction

Historically, noninvasive assessments of gut inflammation have utilized tests such as the detection of fecal white blood cells [1] and whole stool lavage. However, these provide inadequate assessment of gut inflammation. The need to improve assessment of gut inflammation has driven investigation into additional biomarkers of inflammation. Calprotectin, S100A12, and lactoferrin have generally been well described as fecal biomarkers in inflammatory bowel disease (IBD) [2]. However, other less well-established fecal biomarkers include M2-pyruvate kinase, osteoprotegerin, myeloperoxidase, HMGB1, chitinase 3-like 1, defensins, matrix metalloproteinases, and human nucleic acid: most of these have been assessed in single cohorts and require further extensive evaluations and validation. This review will focus on these novel fecal biomarkers, with mention of the future potential of biomarkers in diagnosis, prognosis, and monitoring of disease activity in IBD.

## 2. M2-PK

Pyruvate kinase, a key enzyme in the glycolytic pathway [3], can be present in skeletal muscle, heart or brain as a tetramer (M1), or in undifferentiated and proliferating tissues as a dimer (termed M2-PK) [4, 5]. M2-PK can be measured in serum or stool and is stable in stool for up to two days [6]. Fecal M2-PK concentrations are increased in colorectal carcinoma [7], but also in gut inflammation [3] reflecting increased cell turnover. Although, it is postulated that intestinal epithelial cells may be protected against apoptosis by the upregulation of M2-PK through the Bcl-xl pathway in Crohn's disease (CD) [8].

High levels of M2-PK were documented in 81 adults diagnosed with IBD [3] (Table 1). This cohort was compared to a group of 43 subjects with irritable bowel syndrome (IBS) and 7 with colorectal carcinoma. M2-PK concentrations were higher in patients with ulcerative colitis (UC) or CD than in the controls. Furthermore, higher levels were evident in individuals with active IBD than in those with quiescent disease. In a further study, M2-PK was assessed in 105 adults presenting with undifferentiated gastrointestinal symptoms and 94 healthy controls [9]. The 14 adults subsequently diagnosed with organic diseases (only 10 with IBD) had higher fecal concentrations of M2-PK than those with functional symptoms or the controls. M2-PK measurement provided sensitivity of 67% and specificity of 88% in distinguishing between organic and functional diagnoses.

Fecal M2-PK was assessed in a group of Polish children with IBD [4]. Seventy-five children with UC, 32 with CD, and 35 healthy control children provided stool samples. M2-PK levels were higher in children with IBD, and levels correlated with pediatric Crohn disease activity index (PCDAI [10, 11]) scores in the 32 children with CD. Although mean M2-PK levels were higher in those with active disease, 47% of the children with IBD judged to be in remission also had elevated M2-PK (Table 2(a)).

In a recent Australian study, mean fecal M2-PK levels were also higher in 17 children with active CD than in 21 healthy controls (*p* = 0.0007) [12]. However, M2-PK levels did not correlate with PCDAI scores or serum inflammatory markers. There was no relationship between fecal M2-PK and fecal S100A12 levels in the children with active CD. The children with ileocolonic disease tended to have higher M2-PK concentrations than those with isolated colonic or ileal disease (Table 2(b)).

High fecal M2-PK levels also were demonstrated in children with active UC [13]. M2-PK and three other fecal markers (calprotectin, S100A12, and lactoferrin) were evaluated as indicators of the response to first line medical therapy in 101 children with acute severe UC. M2-PK was found to be superior to the other markers in identifying those who subsequently failed intravenous corticosteroids.

In 2014, Czub et al. [14] directly compared M2-PK and calprotectin in assessing the severity and activity of paediatric IBD. Truelove-Witts score was used to describe disease severity in UC patients, and PCDAI was used to assess CD patients. The performance of M2-PK was described as inferior to calprotectin to identify IBD, UC, and CD from healthy controls. In addition, M2-PK was inferior to calprotectin in identifying UC and CD in remission amongst healthy controls. It was postulated that calprotectin reflects paediatric IBD severity and activity better than M2-PK. However, this is in contradiction of the observation of Roszak et al. [15] who state that M2-PK is a more sensitive marker than calprotectin and lactoferrin in evaluating disease activity in UC or CD (Table 2(b)). Further studies are required in this area to clarify this discrepancy.

## 3. Osteoprotegerin

Osteoprotegerin, also known as osteoprotegrin, (OPG) is a basic glycoprotein that is found either as a 60 kDa monomer or as a 120 kDa dimer. OPG is a cytokine receptor and belongs to the TNF superfamily [16, 17]. OPG can be produced by a wide range of cell types, including osteoblasts, B lymphocytes, dendritic cells, bone marrow stromal cells, epithelial cells, and monocytes/macrophages [17, 18]. OPG production may be regulated by proinflammatory mediators [16, 19, 20]. The cellular sources of OPG are distinct to those of the established inflammatory markers calprotectin, lactoferrin, and S100A12.

OPG has a well-established role in bone turnover. The equilibrium of osteoclast and osteoblast activity is coordinated primarily by the receptor activator of nuclear factor-*κ*B (RANK) and its ligand (RANKL). OPG acts as a decoy receptor for RANK [16, 18, 20–22]. In this role as a decoy receptor, OPG inhibits the differentiation, survival, and function of osteoclasts by competitively blocking the interaction between RANK and RANKL [18] promoting bone formation as a counter regulatory response to factors such as inflammatory cytokines (IL-1, TNF*α*) [23]. This is of importance in IBD where there is an established increased fracture risk associated with the disease [24]. However, the impact of intestinal-derived OPG upon bone loss in the context of IBD is yet to be directly established [25].

OPG also may have a role in IBD pathogenesis, quite separate to the role in bone metabolism. The OPG/RANKL/RANK triad may contribute to mucosal and systemic inflammation [17, 20, 22]. RANK, RANKL, and OPG decrease the functional capacity of dendritic cells (DC) and activated T cells but enhance B cell maturation [26–28].

Recently, a small number of studies have demonstrated that OPG can be a useful marker of inflammation in the context of IBD. Nahidi et al. [27] evaluated OPG in children with CD and control children without evidence of underlying gut disease (Table 1, Table 2(b)). OPG was detected in serum, mucosal biopsies, and in the stool. Levels of OPG were greatly increased in stool samples collected from the children with CD and in the endoscopically obtained mucosal biopsies. In addition, serum levels of OPG were markedly elevated in those children with severe CD compared to control values. Furthermore, serum and fecal levels fell substantially with remission induction therapy in a subset of children (exclusive enteral nutrition in this instance). In this group of children with CD, those children with isolated colonic involvement had greater levels than a group with ileocolonic disease and serum and fecal OPG did not correlate. Although OPG did not correlate with the PCDAI scores, levels did correlate with modified PCDAI scores (includes the serum inflammatory marker components of the PCDAI, with the addition of a score for CRP [29]). In addition, fecal OPG also correlated with fecal S100A12 and serum CRP at diagnosis of IBD, yet did not correlate following treatment.

Galliera et al. [30] measured serum OPG and RANKL levels following the administration of the anti-TNF-*α* inhibitor infliximab in adult patients with IBD. In this study, OPG levels also decreased with treatment, in correlation with falling CRP levels. It is important to note that OPG levels did not fall acutely and were only significantly lower 22 weeks after the commencement of therapy. The authors suggest that in this setting, OPG is representative of the inflammatory response and not bone turnover.

In addition, Sylvester et al. [31] recently investigated whether fecal OPG was able to act as a predictive marker for the treatment of UC in a large group of children. They reported that fecal OPG was elevated in patients who had failed first-line corticosteroid therapy and required infliximab or colectomy. Further, OPG was superior in predicting response to therapy compared to lactoferrin or S100A12. These reports indicate that OPG may be useful in monitoring the inflammatory response in IBD. However, the authors note that OPG is rapidly degraded in stool at room temperature and, therefore, optimal stool collection and storage conditions must be used to accurately assess fecal OPG.

There are, however, conflicting reports on fecal OPG expression. In a preliminary investigation, Skinner et al. [32] report that fecal OPG is elevated at diagnosis of UC but not of CD and, therefore, may have potential as a UC specific marker. This is in contrast to Nahidi et al. [27] who report that OPG is elevated at diagnosis of CD, albeit levels were more elevated with severe disease. Nevertheless, the expression pattern of OPG appears unique amongst the stool inflammatory markers investigated to date. Consequently, OPG has the potential to enhance knowledge of the intestinal inflammatory picture. However, it is clear that further investigation into the settings of OPG expression in the intestinal mucosa is required to advance our understanding of how OPG fits into the inflammatory cascade and its role in the pathogenesis of the inflammatory response in IBD.

## 4. Myeloperoxidase

Myeloperoxidase (MPO) is one of a number of proteins stored in and released from neutrophil secretory granules: it is stable for at least 3 days in stool [33]. One potential limitation of myeloperoxidase as an inflammatory marker is its cationic charge, which may lead it to bind to fecal particles, limiting reliability as a disease marker [34].

MPO levels were greatly increased in a cohort of 55 Indian patients with UC compared to 74 healthy controls (0.42 units versuss 0.06 units: *p* < 0.001) [35] (Table 1). In differentiating between UC and no inflammation, a sensitivity of 89% but specificity of just 51.4% was observed. Levels correlated with endoscopic severity scores and fell following therapeutic intervention. However, levels did not correlate with endoscopic extent or histological severity scores [36] (Table 2(b)).

Sangfelt et al. [37] also showed significant correlation between MPO concentrations and endoscopic and clinical activity in a study of 11 Swedish patients with UC. A Japanese study involving 33 patients with UC and 32 patients with CD again illustrated a strong relationship between MPO and endoscopic extent or histological grade [38] (Table 2(a)). In this instance, MPO levels fell in response to therapy and as suggested earlier fecal MPO may be used as a noninvasive biomarker for the response to treatment [36].

## 5. HMGB1

The nuclear protein, high-mobility group box (HMGB) 1, is released from immune cells in the setting of inflammation. It has been described as an alarmin, with key inflammatory properties.

One study has assessed HMGB1 in a cohort of children with IBD [39]. Fecal levels of HMGB1 protein were greatly increased in 40 children with IBD, whilst being undetectable in 13 healthy controls (Table 1). These authors documented high cytoplasmic levels of HMGB1 in mucosal biopsies, suggesting this to be the source of the increased production. Although likely derived from different cellular sources, fecal HMGB1 correlated with fecal calprotectin (*r* = 0.77). Furthermore, fecal concentrations reflected endoscopic severity in a subset of 16 children with clinically inactive disease but active mucosal findings. These results were reproduced by the same group in adult patients with IBD [39]. However, there was no significant correlation between HMGB1 levels and activity indexes (Crohn's disease activity index (CDAI) and partial Mayo score for UC) (Table 2(a)). More interestingly, there was a significant correlation between fecal HMGBI levels and endoscopic indices (*r* = 0.763, *p* < 0.001 for the Simple endoscopic score for Crohn's disease (SES-CD) and *r* = 0.44, *p* < 0.05 for endoscopic Mayo subscore). Palone et al. [40] therefore hypothesise that fecal HMGB1 could play a role as a marker of subclinical gut inflammation and a novel biomarker of mucosal healing.

HMGB1 has previously been shown to reflect intestinal inflammation in the context of enteric infection [41]. In addition, animal studies have shown that HMGB1 production and signalling can be significantly modulated by therapeutic intervention with dipotassium glycyrrhizate [42] or ethyl pyruvate [43] suggesting that such intervention could have a place in human IBD. Although these data could support HMGB1 having a role in IBD, further assessments are required before HMGB1 could be considered further.

## 6. Chitinase 3-Like 1

Chitinase 3-like 1 (CHI3L1) binds chitin, an abundant polysaccharide. The expression of CHI3L1 is upregulated in various cell types such as colonocytes and lamina propria macrophages [44]. This protein is known to enhance bacterial invasion and adhesion to epithelial cells. Increased serum levels of this protein have been demonstrated in patients with IBD [45].

Aomatsu et al. [46] demonstrated marked elevations of fecal CHI3L1 in 92 children with IBD compared to healthy controls. Using a cut-off of 13.7 ng/g, fecal concentrations were able to distinguish between IBD and control children with sensitivity of 84.7% and specificity of 88.9% (Table 1). In both UC and CD, fecal levels correlated with endoscopic severity scores and also related closely with fecal calprotectin levels (Table 2(a), Table 2(b)). Finally, in a subset of 11 children with paired samples before and after therapy to induce remission, fecal levels of CHI3L1 were noted to fall (*p* = 0.01). In addition to its presumed role in IBD, CHI3L1 was suggested by Chen et al. [47] to play a role in inflammation-associated neoplastic modification in colonic epithelial cells.

Although there is some rationale to consider that this protein may be a useful marker, further assessment and validation is required.

## 7. Human Beta Defensin 2

Defensins are innate antimicrobial peptides that are produced at epithelial borders and contribute to host defence [48]. Several members of the *β* defensin group are produced in the colon by epithelial cells and plasma cells. An initial study completed in French children demonstrated that human *β* defensin (HBD) 2 could be measured in stool samples [49]. Although able to be detected in control children, levels were substantially higher in those with IBD (*p* = 0.0002), especially those with UC (Table 1).

One further study has evaluated fecal levels of HBD2 in a group of adults [50]. HBD2 was elevated in 30 adult patients with UC compared to 24 healthy controls (106.9 versus 29.9: *p* = 0.001) (Table 1). However, levels were also increased in a group of 46 patients with IBS compared to the control group, but not different to those with UC. These results suggest a proinflammatory activation of the mucosal immune system in patients with IBS as well as IBD and suggest that this marker may not be specific to IBD.

## 8. Matrix Metalloproteinase

The matrix metalloproteinases (MMP) are a family of key biological mediators involved in tissue degradation and restitution. A number of studies have examined the roles of MMP proteins in IBD [51, 52]: these include regulation, inflammation, and tissue destruction. Furthermore, the balance between MMPs and their inhibitors (tissue inhibitors of metalloproteinases: TIMPs) may influence fibrosis and stricture development.

MMPs are expressed in areas of inflammation and ulceration in the gut, and several MMPs are overexpressed in IBD [51, 53–57]. MMP-2 has been reported to be elevated in the inflamed tissue from IBD patients [58]. Gao et al. [58] reported increased tissue mRNA levels that correlated with severity of inflammation. Immunohistochemistry studies revealed that MMP-2 was present in the extracellular matrix of the submucosa. Furthermore, elevated serum [59] and urine [60] levels have also been reported in IBD patents.

MMP was also monitored in patients with CD managed with infliximab. Interestingly, MMP-2 serum levels increased, both in responders and nonresponders to treatment, and this was hypothesised to be due to increased intestinal cell turnover [59]. Garg et al. [61] reported the findings of an experimental colitis model using dextran sodium sulphate-induced colitis in MMP-2-ablated mice. In this model, the MMP-2-ablated mice developed a more severe colitis than the control animals indicating that MMP-2 has a protective role against developing colitis and may explain elevated levels in response to treatment.

Most interest in MMPs as disease markers of IBD has focused on MMP-9. Kofla-Dlubacz et al. [62] showed that serum MMP-9 concentrations correlated with CRP levels and with disease activity (using PCDAI scores) in 82 children with CD [63] and in 31 children with UC. Annahazi et al. [64] have subsequently delineated fecal levels in 47 patients with IBD, 23 with IBS, and 24 control patients. In the subjects with UC, MMP-9 concentrations correlated with endoscopic and clinical severity scores (*p* < 0.001 for both relationships) and were also associated with CRP levels (*p* = 0.002). Furthermore, MMP-9 levels correlated with fecal calprotectin (*p* = 0.014).

Despite MMP-9 being elevated in both CD and UC, the performance of MMP-9 as a disease marker appears to be better in UC and pouchitis [65]. Kolho et al. [66] compared the performance of MMP-9 to that of calprotectin in distinguishing IBD from non-IBD subjects. Although MMP-9 performance was comparable to calprotectin in those with UC, MMP-9 was inferior to calprotectin in CD [66]. There are differing reports regarding the response of MMP-9 to therapy. Gao et al. [59] reported that serum MMP-9 levels fell in response to a single dose of infliximab; however, Makitalo et al. [67] reported that serum MMP-9 levels were not altered with therapy. Overall, it appears that MMP-9 may be a useful biomarker in the assessment of undifferentiated gut symptoms and in the evaluation of mucosal inflammatory activity although further work is needed to more fully describe these expression patterns.

Several other MMP's are also elevated in IBD. MMP-7 and MMP-13 mRNA levels were elevated in biopsy specimens from CD and UC patients [68]. Serum MMP-3 [62] and MMP-8 [67] levels are also elevated in IBD. However, the role of MMP as markers in IBD may not be restricted to reporters of inflammation and tissue repair. MMP expression is also associated with colorectal tumors. MMP-7 mRNA had been detected in cancerous intestinal tissue [69] and has been proposed to be involved in the growth of tumors [70]. Therefore, MMP-7 may also have a potential role in colorectal cancer screening, including in the context of colitis-associated cancer. However, fecal evaluation of this marker is not yet described.

## 9. Human Nucleic Acid (DNA and miRNA)

One study has assessed human deoxyribonucleic acid levels in stool of 36 individuals with UC [71]. Excretion of DNA correlated with clinical disease activity and endoscopic severity (Table 2(b)). In differentiating between active disease and remission, this test provided sensitivity of 67% and specificity of 100%. The same group subsequently assessed this biomarker in a cohort of 54 adults with inactive UC [72] (Table 2(a)). Over a 12-month period of observation, 23 of the subjects relapsed. Fecal DNA levels remained stable in those who remained well, whilst levels increased in the patients with relapse, providing a predictive marker.

In addition to DNA, RNA or more specifically microRNA (miRNA) has the potential to be used as a disease marker in IBD. miRNAs are small noncoding RNA molecules that are capable of regulating gene expression at the posttranscriptional level. Expression patterns have been described in intestinal biopsies collected from IBD patients with a number of specific miRNA reported to be upregulated in both CD and UC [73–75]. In CD, it has been documented by Wu et al. [75] that the pattern of expression of miRNA in ileal and colonic biopsies differs significantly, meaning that no miRNA expressions were overlapping. In CD and UC, when compared to healthy controls, specific upregulated miRNA (CD [76], UC [77]) and downregulated miRNA (CD [78], UC [76]) were described. Importantly, differentially expressed miRNA were also detected in the serum of IBD patients indicating the potential of these molecules to be used as disease markers.

However, there are several difficulties that need to be overcome to compare these studies. For example, concurrent medications, the inflammatory status, and the location of the biopsy need to be considered when interpreting the role of miRNAs [79, 80]. Further investigation of the detection of miRNA in stool is required. In addition, the full role of these molecules in IBD is still required; however, differential expression of specific miRNA have been implicated in disease pathogenesis [74].

## 10. Future Potential of Biomarkers in IBD

A biomarker can be described as a product that provides a measurable indication of the presence and/or severity of disease or physiological state of an organism. The holy grail of a biomarker is that it is disease specific, correlates highly with disease severity, and can provide both diagnostic and prognostic indications. To date, several promising fecal biomarkers for IBD have been identified. However, none of the currently described markers are disease specific so the search for better biomarkers of IBD continues. A number of promising novel biomarkers have been identified, and this article has described a portion of the most promising novel markers. However, there are many more potential biomarkers of IBD and further work is required to determine if any of the newly identified markers will be equal to, or surpass, the utility of currently available markers. The aims of the tables are to describe and compare the current knowledge in the use of novel markers: as markers of diagnostic tests for IBD (Table 1), as markers to assess disease activity (inactive versus active) in IBD (Table 2(a)), and finally, as markers of disease severity in IBD (Table 2(b)).

The initial identification of calprotectin as a biomarker of IBD was greeted with great enthusiasm and promise that it would impact clinical care. However, after nearly 30 years of calprotectin research, the incorporation of this biomarker into a routine clinical care has been slow. Calprotectin has not replaced current nonspecific inflammatory markers, but when used, it is generally used as an adjunct disease marker. Therefore, it is reasonable to suspect a long wait for new IBD biomarkers to appear in the clinic. Nevertheless, biomarkers of IBD have a bright future, in research studies at least, as prognostic indicators and in the search for personalised and improved IBD clinical care.

## 11. Conclusion

There also are several promising markers described here that likely reflect different cellular sources and different aspects of the IBD response. Currently, no single marker appears to be sought after highly sensitive, diagnostic, and prognostic IBD specific indicator. However, a number of novel biomarkers have been evaluated in just one or two cohorts: these all require more extensive assessments in various settings to establish their roles in identifying, monitoring, and predicting disease behaviour.

More clinical studies are required to ascertain the full potential of fecal biomarkers in IBD. Although such evaluations should focus on specific biomarkers, comparative assessments are also required, yet there remains plenty of potential for novel biomarkers to impact clinical care.

## Figures and Tables

**Table 1:** Use of novel fecal markers as diagnostic test for IBD.

Ref	Age	Population	*N*	Comparison groups	Cut-off	Sn	Sp	PPV	NPV	*p* value
*M2 pyruvate kinase (M2-PK)*									
[3]	P	HC/CD/UC	142	HC versus IBD	4 U/g	96.2	94.3	96.2	94.3	NA
				HC versus CD	4 U/g	100	94.3	89.5	100	
				HC versus UC	4 U/g	94.3	94.3	94.3	94.3	
				HC versus IBD	5 U/g	94.2	94.3	96.1	91.7	
				HC versus CD	5 U/g	94.1	97.1	88.9	97.1	
				HC versus UC	5 U/g	94.3	97.1	97.1	94.4	
[10]	P	HC/CD	38	HC versus CD						0.0007

*Osteoprotegerin (OPG)*									
[29]	P	HC/CD	127	HC versus CD						<0.0001

*Myeloperoxidase (MPO)*									
[34]	A	HC/UC	74	HC versus UC	0.065 U/ml	89	51	89	51	NA
[36]	A	HC/UC	109	HC versus UC	0.065 U/ml					<0.001
[38]	A	HC/CD/UC	51	HC versus CD						<0.0001
				HC versus UC						<0.0001

*High-mobility group box (HMGB1)*									
[39]	P	HC/CD/UC	54	HC versus CD						<0.001
				HC versus UC						<0.001

*Chitinase 3-like 1 (CHI3L1)*									
[46]	P	HC/CD/UC	237	HC versus IBD	13.7 ng⁄g	84.7	88.9	96.8	59.3	
				HC versus CD		81.6	80	93.9	53.3	
				HC versus UC		88.2	100	100	66.7	

*Human β defensin (HBD) 2*									
[49]	P	HC/CD/IC/UC	46	HC versus IBD						0.0002
				HC versus CD						<0.01
				HC versus IC						<0.01
				HC versus UC						<0.0001
				CD versus UC						0.0063

[50]	A	HC/IBS/UC	100	HC versus IBS						0.032
				HC versus UC						<0.001
				IBS versus UC						0.165

Age: A: adult; P: paediatric; *N*: number of patients; Sn: sensitivity; Sp: specificity; PPV: positive predictive value; NPV: negative predictive value; HC: healthy controls; IBD = CD + UC(+IC); CD: Crohn's disease; UC: ulcerative colitis; IC: inflammatory colitis; NA: not available.

**(a):** 

Ref	Age	Pop.	*N*	Inactive	Active	*p* value	Comments
*M2 pyruvate kinase (M2-PK)*							
[3]	A	CD	31	[5] 0.55 U/ml	[26] 30 U/ml	<0.005	
		UC	50	[13] 1.2 U/ml	[37] 40 U/ml	0.006	
[4]	P	CD	32	[15] 16.5 U/g	[17] 96.3 U/g	NS	
		UC	75	[40] 1.5 U/g	[35] 149 U/g	0.0003	

*Myeloperoxidase (MPO)*							
[38]	A	CD	32	[15]	[18]	<0.001	Active: IOIBD score ≥ 2
		UC	33	[14]	[18]	<0.001	Truelove and Witts' severity index

*High-mobility group box (HMGB1)*							
[39]	P	CD	19	[8]	[11]	NS	Active: PCDAI > 10
		UC	21	[8]	[13]	NS	Active: PUCAI ≥ 10

*Chitinase 3-like 1 (CHI3L1)*							
[46]	P	CD	87	[39] 18.4 ng/g	[48] 632.7 ng/g	<0.01	Active: PCDAI ≥ 10
		UC	94	[50] 15.8 ng/g	[44] 366.6 ng/g	<0.01	Active: PUCAI ≥ 10

*Human nucleic acid*							
[72]	A	UC	54	3.9 copies/*μ*g	259.0 copies/*μ*g	*<*0.01	Active: colitis activity index ≥ 7

**(b):** 

Ref	Age	Pop.	*N*	*r*	*p* value	Disease severity
*M2 pyruvate kinase (M2-PK)*						
[12]	P	CD	17	NA	NS	PCDAI
[15]	P	CD	47	0.82	<0.05	PCDAI
		UC	37	0.77	<0.05	Truelove and Witts' severity index

*Osteoprotegerin (OPG)*						
[27]	P	CD	82	0.15	0.31	PCDAI
				0.16	0.21	Modified PCDAI
				0.10	0.22	PCDAI with logged OPG
				0.35	0.0001	Modified PCDAI with logged OPG

*Myeloperoxidase (MPO)*						
[36]	A	UC	55	NA	0.02	Endoscopy severity
				NA	NS	Endoscopic extent or histology score

*Chitinase 3-like 1 (CHI3L1)*						
[46]	P	CD	25	0.61	<0.01	SES-CD scoring system
				0.49	<0.01	PCDAI
		UC	42	0.73	<0.01	Sum of the Matts' score
				0.47	<0.01	PUCAI

*Human nucleic acid*						
[71]	A	UC	31	0.59	<0.05	Clinical index of Rachmilewitz
				0.76	<0.01	Endoscopic score

Age: A: adult; P: paediatric, values are median; Pop.: type of population; *N*: number of patients; NS: nonsignificant; square brackets : number of patients, *r*: correlation coefficient; CD: Crohn's disease; UC: ulcerative colitis; IOIBD: International Organization for the Study of Inflammatory Bowel Diseases score; PCDAI: paediatric Crohn's disease activity index; PUCAI: paediatric ulcerative colitis activity index; SES-CD: simple endoscopic score for Crohn's disease.
